# Using an exon microarray to identify a global profile of gene expression and alternative splicing in K562 cells exposed to sodium valproate

**DOI:** 10.3892/or.2011.1601

**Published:** 2011-12-21

**Authors:** XIANG-ZHONG ZHANG, AI-HUA YIN, XIAO-YU ZHU, QIAN DING, CHUN-HUAI WANG, YUN-XIAN CHEN

**Affiliations:** 1 Department of Hematology, The First Affiliated Hospital of Sun Yat-sen University, Guangzhou, Guangdong 510080; 2 The Clinical Genetic Centre, Guangdong Women and Children Hospital, Guangzhou, Guangdong 510010; 3 Department of Hematology, Anhui Provincial Hospital Affiliated to Anhui Medical University, Hefei, Anhui 23000; 4 Department of Hematology, Guizhou Provincial Hospital, Guiyang, Guizhou 550002, P.R. China

**Keywords:** chronic myelogenous leukaemia, sodium valproate, exon microarray, gene expression profile, alternative splicing

## Abstract

To investigate the effect of valproate treatment on the K562 cell line, a model for chronic myelogenous leukaemia, the growth and survival of the K562 cell line were investigated using the Annexin-V/PI dual staining method, and global profiles of gene expression and alternative splicing in K562 cells were assessed using exon microarrays. A significant increase in cell apoptosis was observed in valproate-exposed K562 cells using flow cytometry. A total of 628 transcripts were identified as being significantly differentially expressed. The number of genes demonstrating increased expression levels was greater than the number of genes demonstrating decreased expression levels (445 genes vs. 183 genes, respectively). The significant enrichment analysis of GO terms for the differentially expressed genes revealed that these genes are involved in many important biological processes such as apoptosis. Six of the genes observed to be differentially expressed that might be involved in apoptosis were selected to undergo qRT-PCR validation. In total, 198 candidates of alternative splicing variants were identified. Among them, three alternative splicing events were selected for validation, and CBLC and TBX1 were confirmed to be alternatively spliced by semi-nested PCR. In conclusion, valproate exposure facilitated cell apoptosis, altered mRNA expression and alternative splicing events in the K562 cell line.

## Introduction

Recently, the anti-epileptic drug valproate, also used for the treatment of bipolar disorder, migraine and neuropathic pain, has attracted increased interest for its use in cancer therapy. Many clinical studies have shown that VPA, alone or in combination with other non-classical anti-leukaemic compounds, exerts significant anti-leukaemic effects in patients with chronic myeloid leukaemia (CML). Studies have also demonstrated that VPA inhibits histone deacetylases (HDAC), modulates cell cycle, induces tumour cell death and inhibits angiogenesis in various tumour models. The exact molecular mechanisms underlying the observed anti-CML effect, however, remain to be elucidated.

Cancer formation and persistence may not only be a result of genetic mutations, but may also be due to changes in the patterns of epigenetic modifications. Various epigenetic factors interact with each other to co-regulate gene expression, including the alternative splicing (AS) of mRNA. These processes mediate the progression of cancer. The most common epigenetic modifications include the methylation of CpG islands within the DNA and the modification of amino acids in the N-terminal histone tails, particularly reversible histone acetylation. Accumulating sources of evidence indicate that AS is altered or becomes aberrant during the progression of CML. For example, unusual splice variants of several genes, including BCR-ABL1, Ikaros, osteocalcin (OCN), growth factor independent 1b (Gfi1b) and c-Fes, have been detected in CML cell lines and or tumour tissues (1–5). These observed changes may potentially be a result of epigenetic modifications. Recently, DNA methylation and histone acetylation status of 5′ sequences of CD7 and ELA2, genes associated with CML clinical outcome, in leukaemic cell lines and primitive leukaemic cells from chronic phase CML patients have been reported to contribute to the variable expression of these two genes (6).

VPA exposure, similar to other HDAC inhibitors, results in the altered expression of many genes and the switching of splice sites in certain diseases by promoting histone acetylation and/or inducing demethylation (7–9). For example, VPA was found to trigger DNA demethylation and restore the splicing pattern of SMN2 transcripts in fibroblast cultures derived from proximal spinal muscular atrophy (SMA) patients via Htra2-β1, the splicing factor essential for the inclusion of exon 7 into SMN2 mRNA (10). Since AS studies have traditionally focused on single genes, little is known about genome-wide shifting of splicing sites during the progression of CML in a controlled setting or following VPA treatment. Recently, exon microarrays have been successfully used to evaluate genome-wide splicing events in other cancer types (11,12). Simultaneous detections of the expression profiling and splicing events based on the same exon microarray have also been reported (13,14). In the current study, we investigated the effect of VPA treatment on the K562 cell line, a model for CML, at the cell level using the Annexin-V/PI dual staining method and genome-wide expression and AS levels using exon microarray.

## Materials and methods

### Culture of K562 cell line

K562, a human chronic myelogenious leukemic cell line was obtained from the Sun Yat-sen University Cancer Center. K562 cells were grown and maintained in RPMI-1640 (Life Technology Corp., Camarillo, CA) containing 15% FBS, 100 U/ml penicillin and 1 mg/ml streptomycin in a 5% CO_2_ incubator at 37°C.

### Assessment of apoptosis by Annexin-V/PI dual staining method

Apoptotic cells were determined by fluorescence-activated cell sorting (FACS) analysis after staining with Annexin-V/PI. Briefly, K562 cells were exposed to 2 mM of VPA for 48 h. The control group received drug-free medium. At the end of the treatment period, the control (untreated) and treated cells were harvested, washed twice with cold PBS at 1,000 rpm for 5 min and passed through a 400-mesh cell strainer. Then cells were collected and gently resuspended to a final concentration of 1×10^6^/ml in binding buffer. Annexin-V-FITC (5 μl) and 10 μl 50 mg/l PI were added to 100 μl of cell suspension and incubated with cells in the dark for 15 min. At the end of incubation, 400 μl binding buffer were added and analysis by a FACScan flow cytometer was carried out to discriminate between live and apoptotic cells.

### Analysis of gene expression profile and AS by the Agilent human exon microarray

K562 cells were either treated with 2 mM VPA for 12 h or left untreated as a control. RNA was extracted from cultured cells using the RNeasy Mini Kit (Qiagen, USA) according to the manufacturer’s instructions. RNA quality was assessed using formaldehyde agarose gel electrophoresis and was quantified via spectrophotometry (Nanodrop, Wilmington, DE). RNA was amplified using the Agilent Low Input Quick Amp WT labeling kit according to the manufacturer’s guidelines with a small modification. Briefly, 100 ng of total RNA was used to synthesise double-stranded cDNA. RNA was amplified by *in vitro* transcription using WT Primer mix that reverse transcribed throughout the 3′ to 5′ end of mRNA, replacing the T7 promoter primer using in standard expression array, in which only the 3′ end of mRNA was reverse-transcribed. aRNA was then reverse transcribed into cDNA and was labelled with cy3-dCTP or cy5-dCTP using Klenow enzyme. Fluorescent dye-labelled cDNA was hybridised to an Agilent Human 4×180K Exon Microarray, which included 174,458 exon probes, targeting 20,411 genes. Hybridisation, scanning and washing were performed on Agilent’s Microarray Platform according to Agilent’s standard protocols. The array data were analysed with Agilent Feature Extraction software. Following global mean normalization, probes with an intensity <400 were removed for further analysis. The geometric mean of all exon-level probe signalling for each transcript was considered the transcript-level probe signalling of this transcript. Based on the evaluation of the systematic noise of microarray experiments using self-to-self comparisons (15), we defined differentially expressed genes as genes with at least a 2-fold change in transcript-level probe signalling in VPA-treated samples relative to those observed in control samples.

Differentially expressed genes were further analysed based on a significant enrichment of GO terms using hypergeometric distribution in the R language package software.

Detection of AS genes was based on the ‘Splicing Index (SI)’ model (16), which aimed to identify exons that have differential inclusion rates (relative to the gene level) between two sample groups.

### Validation of differentially expressed genes and AS genes

From the differentially expressed genes identified by microarray analysis, six genes were selected based on the function of interest and expression levels were confirmed using quantitative real-time RT-PCR. One microgram of DNase-treated total RNA, isolated from K562 cell line treated with or without valproate exclusively for validation of differentially expressed genes and AS genes, were reverse transcribed with oligo(dT)15 using M-MLV reverse transcriptase (Life Technologies) in a total volume of 20 μl reaction volume. Following reverse transcription reaction, 1 μl of this mixture was employed for a qPCR program of 45 cycles of melting (30 sec at 94°C), annealing (30 sec at 58°C) and extension (30 sec at 72°C). The 20 μl reaction mixture contained 1X PCR Buffer (Mg^2+^ Plus), 200 μM of each dNTP, 0.5 μM of forward primer, 0.5 μM of reverse primer (Table I), and EvaGreen Master Mix in a LightCycler^®^ 480 Real-Time PCR System (Roche Applied Science). Data were analysed by the 2^−ΔΔCt^ method (17) using glyceraldehyde 3-phosphate dehydrogenase (GADPH) as a reference gene. All other results are shown as fold-change relative to GADPH control.

Alternative forms of genes are often found to be expressed at lower levels than constitutive forms. In such cases, traditional flanking-PCR, in which primers target constant exon sequences flanking each alternative exon, results in a poor sensitivity for detecting alternative variants. To validate the alternatively spliced genes detected by the exon array, we conducted semi-nested PCR analyses using reverse primers that target the predicted exon rather than the constitutive exon and that were designed specifically for two rounds of PCR, as described by Leparc and Mitra (18). Briefly, 1 μg of DNase-treated total RNA, isolated from the K562 cell line treated with or without valproate exclusively for validation of differentially expressed genes and AS genes, was reverse transcribed with random hexanucleotide primers using M-MLV reverse transcriptase in a total reaction volume of 20 μl. Following reverse transcription, 1 μl of this cDNA mixture was used for a first round program of 25 cycles of melting (30 sec at 94°C), annealing (30 sec at 55°C) and extension (30 sec at 72°C). The 20 μl reaction mixture contained 1X PCR Buffer (Mg^2+^ Plus), 200 μM of each dNTP, 0.5 μM of forward primer, 0.5 μM of reverse primer (primer sequences are provided in Table II), 0.5 unit of Takara Taq HS and 1.0 μl of cDNA. The second round reaction was performed for 30 cycles using the same program but with a 1:100 dilution of first round reaction as the template. PCR products were separated on 2% agarose gels supplemented with ethidium bromide. DNA was visualised under a UV light.

### Statistics

The Student’s t-test (Microsoft Excel, Microsoft Corp., Seattle, WA) was performed to determine the significance between groups. A P-value of <0.05 was considered statistically significant. The microarray data have been deposited in NCBI’s Gene Expression Omnibus (http://www.ncbi.nlm.nih.gov/geo) and are accessible through GEO series accession number GSE 38252.

## Results

### Analysis of apoptosis using the Annexin-V/PI dual staining method

**Figure 1:**
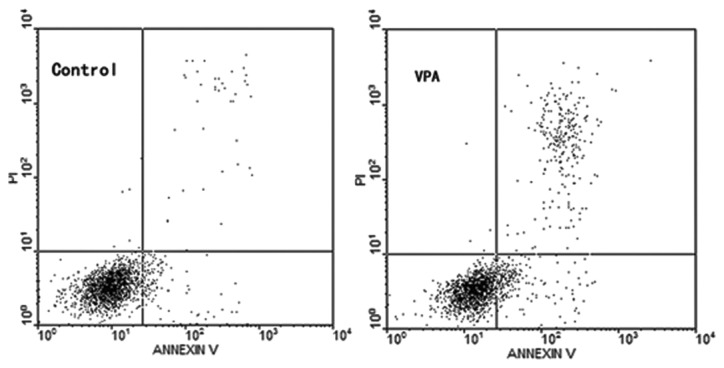
Flow cytometry analysis of apoptosis induced by exposure to 2 mM of VPA for 48 h in K562 cells using Annexin-V/PI.

Control and treated K562 cells were stained with Annexin-V-FITC/PI and gated into LR (lower right) and UR (upper right) quadrants. Cells in the LR and UR quadrants were considered early apoptotic (Annexin^+^/PI^−^) and late apoptotic (Annexin^+^/PI^+^), respectively (Fig. 1). The extent of apoptosis was expressed as the sum of the percentage of cells gated in LR and UR quadrants. Relative to the K562 control, a significant increase in apoptosis was observed in K562 cells exposed to 2 mM VPA for 48 h (Table III).

### Differentially expressed genes in K562 cells pretreated with VPA detected by Agilent human exon microarray

Differentially expressed genes are more easily detected in cells during the primary response phase of a drug exposure at certain doses that result in a 10–20% decrease in cell viability. Based on this information, we pretreated K562 cells with 2 mM VPA for 12 h, a dose resulting in an approximately 12% decrease in cell viability. The Agilent Human 4×180K Exon Microarray, with probes targeting all exons within genes, can provide gene-level expression information as well as AS information. Using this Agilent microarray platform, the transcription profiles of K562 cells treated with or without VPA were analysed. A total of 628 transcripts were identified as being significantly differentially expressed (full list of differentially expressed genes is available upon request). The number of genes demonstrating increased expression levels was greater than the number of genes demonstrating decreased expression levels (445 vs. 183 genes, respectively).

The significant enrichment analysis of GO terms for the differentially expressed genes using the R language package software revealed that these genes are involved in many important biological processes, including apoptosis and the regulation of B cell differentiation. As shown in Table IV, there were five differentially expressed genes involved in the positive regulation of anti-apoptosis and three differentially expressed genes involved in the induction of apoptosis by intracellular signals.

### Validation of the differentially expressed genes using qRT-PCR

**Figure 2:**
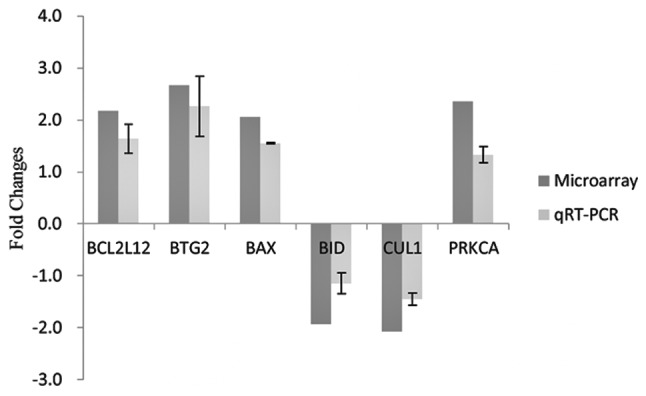
Alterations in the expression levels identified by microarray were confirmed using qRT-PCR. Fold-changes between K562 cells treated with and without vaproate detected by microarray were compared with those measured by qRT-PCR. In the qRT-PCR assay, RNA was isolated from the K562 cell line treated with or without valproate exclusively for validation of differentially expressed genes and AS genes. mRNA levels were normalized with GADPH and fold-changes were calculated by dividing the mRNA levels of each K563 cell line treated with vaproate by mean mRNA levels from control samples in triplicate. Data are mean values ± standard deviations of the means from three independent experiments performed in triplicate.

Based on the microarray results, the differentially expressed genes with high probe signals in the categories of positive regulation of anti-apoptosis and induction of apoptosis by intracellular signals were selected for qRT-PCR confirmation. As depicted in Fig. 2 and consistent with the results obtained from microarray analysis, BCL2L12, BTG2, BAX and PRKCA were upregulated and CUL1 was downregulated.

### AS events in K562 cells pretreated with VPA detected by Agilent human exon microarray

In the present study, differentially expressed splice variants in K562 cells treated with VPA were evaluated using an Agilent Human 4×180K Exon Microarray designed to detect differential splicing of 20,411 genes (174,458 exon probes). In total, 198 candidates of AS variants were identified (full list of candidate AS variants is available upon request). The significant enrichment analysis of GO terms for the AS genes using the R language package software revealed that the AS genes were involved in many important biological processes, including negative regulation of the cytokine-mediated signalling pathway, negative regulation of erythrocyte differentiation, positive regulation of γ-δ T cell differentiation, mitotic cell cycle spindle assembly checkpoint, positive regulation of neuron apoptosis, DNA damage checkpoint and the Wnt receptor signalling pathway (Table V).

**Figure 3:**
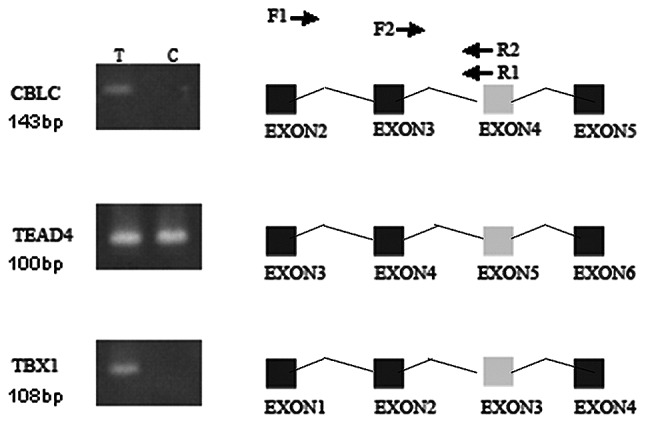
Validation of alternative splicing using a semi-nested PCR assay. Total RNA, isolated from K562 cell line treated with or without valproate exclusively for validation of differentially expressed genes and AS genes, was reverse transcribed with random hexanucleotide primers using M-MLV reverse transcriptase. Following reverse transcription, two rounds of PCR were performed with reverse primers that target the predicted exon rather than the constitutive exon.

After filtering and inspecting the results in order to characterise the candidate splicing events, CBLC, TEAD4 and TBX1 were selected for validation by semi-nested PCR. Events for CBLC and TBX1 were confirmed (Fig. 3).

## Discussion

In this current study, we first investigated the effects of VPA treatment on growth and survival of K562 cells. VPA is a short-chain, branched fatty acid that has recently been described as a potent inhibitor of HDAC at therapeutic concentrations. Unlike other HDAC inhibitors, which are associated with various toxic side effects, VPA is clinically available. It can be taken orally, can cross the blood-brain barrier and can be used for extended periods. HDAC inhibition is responsible for the acetylation of histones, a process mediated by HATs. This event has been associated with the induction of apoptosis in many models of leukaemia (19,20). Thus, the use of VPA prior to or concurrently with anticancer drugs may prove to be a beneficial treatment of leukaemia (21,22). The K562 cell line, originating from a patient diagnosed with CML in terminal blast crisis, is highly undifferentiated and of the granulocytic series. Our data demonstrate that VPA exposure induces cell death in K562 cells in a dose-dependent manner. This result is in agreement with previous reports on CML (23,24) and other types of leukaemia (25,26).

Microarrays allow for the analysis of global gene expression profiles in a single experiment. This assay also provides a high-throughput tool to disclose the molecular mechanisms of anti-leukaemic activity observed following exposure to certain compounds. Based on this high-throughput technology, the underlying molecular mechanisms for the observed anti-leukaemic activity of VPA in CLL cells (27) and AML cells (25,28) have been described. Genome-wide gene expression changes in CML, however, have not been reported. CML is a type of myeloproliferative disease associated with a characteristic chromosomal translocation called the Philadelphia chromosome. Conversely, CLL affects B cell lymphocytes, and AML is characterized by the rapid growth of abnormal white blood cells that accumulate in the bone marrow and interfere with the production of normal blood cells. The observed clinical features and pathology of CML patients, as well as the therapy options, are quite different from that of CLL and AML patients. Consequently, the effect of VPA treatment on CML patients and the underlying molecular mechanisms may be different from effects observed in CLL and AML patients. Based on this information, we investigated the global gene expression profile of K562 cells exposed to VPA using an exon microarray. Our microarray data demonstrated that VPA exposure resulted in widespread gene expression changes in this cell line. We observed a greater number of upregulated genes than downregulated genes (445 genes vs. 173 genes, respectively). Studies have demonstrated that histone acetylation leads to the relaxation of the nucleosome structure, releasing the DNA to allow for transcription. Inhibition of HDAC promotes decondensed chromatin formation, thereby promoting the expression of genes; therefore, our observation that the number of upregulated genes was greater than that of downregulated genes is consistent with VPA acting as an HDAC inhibitor. Among these differentially expressed genes, two functional categories of these genes are of particular interest because the differential expression may indicate the mechanism underlying VPA-induced apoptosis in K562 cells. Based on this data, six of these genes were selected to undergo qRT-PCR validation. BCL2L12, BTG2, BAX and PRKCA were confirmed to be upregulated following VPA exposure, and CUL1 was downregulated. These results suggest that VPA exposure induces K562 cells apoptosis by interfering with genes involved in apoptosis, including both the anti-apoptotic and pro-apoptotic pathways.

AS is a mechanism that increases transcriptome and proteome diversity by allowing for the generation of multiple mRNA products from a single gene. This process is a vast source of biological regulation and diversity that is mis-regulated in cancer (29–31). Antagonistic splice variants of genes involved in apoptosis often exist in a delicate equilibrium, which is perturbed in tumours (32,33). To gain a better understanding of the anti-cancer and pro-apoptotic mechanisms of VPA exposure in a CML model, we used an exon microarray to further investigate the genome-wide shifting of splicing sites in K562 cells treated with or without VPA. In total, 198 candidates of AS variants were identified. CBLC and TBX1 were confirmed as AS by semi-nested PCR. It is interesting to note that VPA-exposed K562 cells exhibited increased AS variants for CBLC and TBX1 than normal (untreated) K562 cells. Studies have demonstrated that cancerous tissues exhibit lower levels of AS than normal tissues (30,34). We hypothesise that CML may result in a loss of AS events and that VPA exposure may restore these lost events.

CBLC is phosphorylated upon activation of a variety of receptors that signal via protein tyrosine kinases. This process is involved in many important molecular pathways, including the ErbB signalling pathway, ubiquitin-mediated proteolysis, endocytosis, the Jak-STAT signalling pathway, the T cell receptor signalling pathway and CML. Mutations in the Cbl family RING finger domain or linker sequence constitute important pathogenic lesions associated with not only preleukaemic CMML, juvenile myelomonocytic leukeamia (JMML), and other myeloproliferative neoplasms (MPN), but also progression to AML, suggesting that impaired degradation of activated tyrosine kinases constitutes an important cancer mechanism (35). CBLC has two splice isoforms. In this study, we detected the long (CBLC-L) and short forms of CBLC (CBLC-S) in VPA-exposed K562 cells, while only CBLC-S was detected in normal K562 cells. Currently, it is unclear whether CBLC-L has a therapeutic benefit in CML patients or whether it is merely a marker of therapeutic improvement in CML patients. Previous study has demonstrated that CBLC-L, but not CBLC-S, is involved in MAPK signalling (36). We hypothesise that the presence of CBLC-L in VPA-exposed K562 cells may be associated with VPA-induced apoptosis.

TBX1 is a member of a phylogenetically conserved family of genes that share a common DNA-binding domain, the T-box. T-box genes encode transcription factors involved in the regulation of developmental processes. Studies using mouse models of DiGeorge syndrome suggest a major role for this gene in the molecular etiology of DGS/VCFS. Currently, only three isoforms of AS (NM_005992.1, NM_080646.1 and NM_080647.1) have been described for this gene, and they all contain exon 3. In this study, we detected an aberrant variant that lacked exon 3 in normal K562 cells. It remains to be elucidated whether the presence of this aberrant variant occurred only after cells have been transformed or whether this form plays a role in the initiation of tumourigenesis. The emergence of an AS variant containing exon 3 in VPA-exposed K562 cells, however, seems to suggest that VPA exposure restores the aberrant AS in K562 cells.

The significant enrichment analysis of GO terms revealed that the differentially expressed genes were involved in the regulation of B cell differentiation, and the AS genes were involved in the positive regulation of γ-δ T cell differentiation. These results suggest that in addition to inducing apoptosis, VPA exposure may also modulate immune responses to CML.

In conclusion, we observed that VPA exposure altered mRNA expression and AS events in the K562 cell line. These alterations might be associated with the pro-apoptotic effect of VPA. The data obtained in this study may provide the basis for further studies to elucidate the molecular and therapeutic potential of VPA in leukaemia treatment.

## Figures and Tables

**Table I:** Primer pairs for qRT-PCR validation of the differentially expressed genes.

Gene symbol	GenBank accession no.	Primer (5′→3′)
BCL2L12	NM_138639	F: TCTCCTGTTCCAACTCCACCTA R: CGCAGTATGGCTTCCTTCTCT
BTG2	NM_006763	F: CCAAACACTCTCCCTACCCATT R: GAGACCATGAGGCTGCTTCTAA
BAX	NM_138764	F: TTCTGACGGCAACTTCAACTG R: TCTTCTTCCAGATGGTGAGTGAG
BID	NM_197966	F: GGTCTGCTGTTCCAGTGGTAA R: TGAGTCCGTCAGTGCCCTTA
CUL1	NM_003592	F:TGGAGCGAGTGGATGGTGAA R: AATGGCGTTGGTGTCCGTATT
PRKCA	NM_002737	F: GGCACACCAGACAATCGTAATC R: GAGGCAGGAGAATCGCTTGAA

**Table II:** Primer pairs for semi-nested PCR validation of alternatively spliced genes.

Gene symbol	GenBank accession no.	Primer (5′→3′)
CBLC	NM_012116_exon 2	F1: ACCACCATTGACCTCACCTG
	NM_012116_ exon 4	R1:TTTGTTGGCAGGGATGGTCT
	NM_012116_ exon 3	F2: ATGAGGTCCAAGAGCGTCTG
	NM_012116_ exon 4	R2: TTTGTTGGCAGGGATGGTCT
TEAD4	NM_003213_ exon 3	F1: AGCTGATTGCCCGCTACATC
	NM_003213_ exon 5	R1: GGCCATGCTACTGTGGAAGG
	NM_003213_ exon 4	F2: GCTAAAGGACCAGGCAGCTAA
	NM_003213_ exon 5	R2: GGCCATGCTACTGTGGAAGG
TBX1	NM_005992_ exon 1	F1: CACTTCAGCACCGTCACCA
	NM_005992_ exon 3	R1: ACGAAGTCCATGAGCAGCATAT
	NM_005992_ exon 2	F2: CACCGAGATGATCGTCACCAA
	NM_005992_ exon 3	R2: ACGAAGTCCATGAGCAGCATAT

**Table III:** Apoptosis rate of K562 cells due to exposure of 2 mM VPA for 48 h.

Groups	n	Percentage of apoptosis (%)
VPA treatment	3	11.47±0.25^a^
Control	3	4.77±0.40

a Compared with the control group, P<0.05.

**Table IV:** Significant enrichment analysis of GO terms of the differentially expressed genes using the R language package software.

Pathways		Genes		
	
Name	P-value^a^	Name	Fold-change	Gene description
Positive regulation of anti-apoptosis	0.0011	CDKN1A	2.03	Cyclin-dependent kinase inhibitor 1A
		IL6ST	3.40	Interleukin 6 signal transducer
		LIFR	2.84	Leukaemia inhibitory factor receptor α
		BTG2	2.68	B-cell translocation gene 2
		CAV1	7.99	Caveolae protein
Regulation of B cell differentiation	0.0047	CD24	4.50	CD24 antigen
		PTPRC	−2.23^b^	Protein tyrosine phosphatase, receptor type, c polypeptide
Leukaemia inhibitory factor signalling pathway	0.0047	IL6ST	3.40	Interleukin 6 signal transducer
		LIFR	2.85	Leukaemia inhibitory factor receptor α1
Induction of apoptosis by intracellular signals	0.0067	CD24	4.50	CD24 antigen
		CDKN1A	2.03	Cyclin-dependent kinase inhibitor 1A
		CUL4A	−2.12	Cullin 4A
Negative regulation of cytokine-mediated signalling pathway	0.0077	PTPRC	−2.23	Protein tyrosine phosphatase, receptor type, c polypeptide
		CAV1	7.99	Caveolae protein
Collagen biosynthetic process	0.0077	COL1A1	2.19	Collagen, type I, α1
		SERPINH1	−2.63	Serpin peptidase inhibitor, clade H, member 1

a P-value for enrichment;

b indicates downregulation.

**Table V:** Significant enrichment analysis of GO terms of the alternative splicing genes using the R language package software.

Pathways		Genes		
	
Name	q-value	Name	SI	Gene description
L-alanine transport	0.018	SLC38A3	0.26	Solute carrier family 38, member 3
		SLC36A1	0.31	Solute carrier family 36 member 1
Negative regulation of cytokine-mediated signaling pathway	0.019	PTPRC	4.91	Protein tyrosine phosphatase, receptor type, C
		PTPRF	3.12	Protein tyrosine phosphatase, receptor type, F
Negative regulation of erythrocyte differentiation	0.019	STAT5A	3.40	Signal transducer and activator of transcription
		LDB1	5.86	LIM domain binding 1 isoform 3
Positive regulation of γ-δ T cell differentiation	0.022	PTPRC	4.91	Protein tyrosine phosphatase, receptor type, C
		STAT5A	3.40	Signal transducer and activator of transcription
Mitotic cell cycle spindle assembly checkpoint	0.042	CENPF	3.55	Centromere protein F
		ATM	53.02	Ataxia telangiectasia mutated isoform 1
Regulation of Rab GTPase activity	0.042	TBC1D10C	0.26	TBC1 domain family, member 10C
		TBC1D2	3.74	TBC1 domain family, member 2
		TBC1D15	40.77	TBC1 domain family, member 15 isoform 1
Positive regulation of neuron apoptosis	0.042	ATM	53.02	Ataxia telangiectasia mutated isoform 1
		PTPRF	3.12	Protein tyrosine phosphatase, receptor type, F
DNA damage checkpoint	0.042	ATM	53.02	Ataxia telangiectasia mutated isoform 1
		BRIP1	69.68	BRCA1 interacting protein C-terminal helicase 1
Wnt receptor signaling pathway, calcium modulating pathway	0.042	ROR2	3.00	Receptor tyrosine kinase-like orphan receptor 2
		WNT11	4.05	Wingless-type MMTV integration site family
Lactation	0.042	STAT5A	3.40	Signal transducer and activator of transcription
		USF2	4.20	Upstream stimulatory factor 2 isoform 1

SI, splicing index. SI is the difference between the Gene Normalized Probe Normalized (GNPN) values between samples treated with and without valproate. The GNPN value is the relative intensity resulting from the division of the probe-normalized value by the geometric mean of probe normalized values of all probes mapping to that gene.

## Abbreviations

CML: chronic myelogenous leukaemia
VPA: sodium valproate
AS: alternative splicing
